# Characterization of thermal storage stability of waste plastic pyrolytic char modified asphalt binders with sulfur

**DOI:** 10.1371/journal.pone.0248465

**Published:** 2021-03-15

**Authors:** Abhinay Kumar, Rajan Choudhary, Ankush Kumar

**Affiliations:** Department of Civil Engineering, Indian Institute of Technology Guwahati, Guwahati, Assam, India; Beijing University of Technology, CHINA

## Abstract

Pyrolysis has gained a strong interest in recent times for sustainable treatment and recovery of energy-rich products from different wastes including plastic. Waste plastic pyrolytic char (PPC) generated as a carbonaceous by-product in the pyrolysis process, is gaining attention as an asphalt binder modifier. Adequate thermal storage stability is an essential requirement for a modified asphalt binder to ensure that the composite offers integrity and homogeneous properties during its storage, handling and transportation in the field. The objective of this study was to evaluate and characterize the thermal storage stability properties of PPC modified binders. PPC modified asphalt binders were fabricated and evaluated at multiple dosages of sulfur as a cross-linking agent. In addition to the conventionally used softening point difference (SPD), characterization of thermal storage stability was attempted using rheology-based separation indices (SIs) derived through temperature sweep, frequency sweep, and multiple stress creep and recovery (MSCR) tests. These rheological SIs were based on complex modulus (G*), Superpave rutting parameter (G*/sin δ), Shenoy rutting parameter (SRP), zero shear viscosity (ZSV), and MSCR J_nr_ (at three stress levels 0.1, 3.2 and 10 kPa). Two formulations of each rheology-based separation index were studied: (1) ratio, and (2) maximum-average difference formulations. The temperature and frequency dependencies of rheological SIs were also evaluated. Further, the Fourier transform infrared spectroscopy (FTIR) was used to characterize storage stability by comparing the chemical functionalities of the PPC modified binders. A 0.3% dosage of sulfur was found to produce the best results considering all SPD, rheology-based SIs and FTIR. Principal component analysis showed that the ratio and maximum-average formulations had similar contributions to the first principal component accounting for more than 99% of the variability.

## Introduction

Effective and sustainable treatment of plastic waste is one of the most persistent and pervasive modern world problems. The generation of plastic solid waste has seen rapid growth ever since the first large-scale plastic production began dating back to 1940s [1]. A quantity of 242 million tonnes was the 2016 estimate for the global generation of plastic waste, accounting for 12 percent of the municipal solid waste stream [2]. If the current waste generation trajectory does not change, the world would be dealing with 460 million tonnes of plastic waste by the year 2030 [3]. The plastic waste crisis has motivated attempts toward using processes that boost a circular plastic economy, and pyrolysis technologies have gained significant interest in this direction. As opposed to a linear concept of ‘take-make-consume-discard’ [4], pyrolysis technologies convert the plastic material into simpler monomers/oligomers and thus support a circular economy [5,6]. By subjecting it to low (or nil) oxygen environment under high temperatures (typically ranging from 300–900°C), pyrolysis converts the plastic waste to liquid oil, gases, and solid pyrolytic char [7]. The yield of pyrolytic products depends on factors such as the design of the reactor, temperature, heating rate, retention time, composition and size of the feedstock, and use of catalyst.

The pyrolysis reaction parameters are customized to maximize the liquid oil yield that has the potential for energy-related uses such as electricity generation and a heat source [7], whereas the solid char is generally regarded a by-product [7–9]. The plastic pyrolytic char (PPC) is obtained as a solid residue at the bottom of the pyrolysis reactor at the conclusion of the pyrolysis process [10,11]. PPC is produced as a by-product of the pyrolysis process and currently finds no large-scale application. It is either disposed of in waste dumping sites or supplied to coal processing units for direct burning, as per the information shared from the personnel of the pyrolysis plant. To maximize the circularity of pyrolysis technology as a sustainable plastic waste treatment method, it is needed to find large-scale applications for the PPC. Some recent studies have reported the use of PPC, a carbonaceous product, as asphalt binder modifier [12,13]. The incorporation of PPC in asphalt modification has also stimulus from the encouraging results reported with chars from the pyrolysis of other materials such as biomasses and end-of-life tires [13–17]. Use of PPC for asphalt binder modification is quite new and requires detailed investigations.

PPC and asphalt binder are two naturally different materials with physical and chemical dissimilarities in density, polarity, solubility, molecular weight and structure. PPC modified asphalts may thus have challenges in terms of storage instability. Adequate thermal storage stability is an essential requirement for a modified asphalt binder to ensure that the composite offers integrity and homogeneous properties during its storage, handling and transportation in the field. Poor storage stability leads to inconsistent composition and rheology of binders between the top and bottom portions of a storage facility and therefore may lead to poor performance against principal pavement distresses such as rutting (at high-service temperatures), fatigue cracking (at intermediate-service temperatures), and thermal cracking (at low-service temperatures) [18–20]. Zani et al. [19] further demonstrated that storage separation negatively affected the fatigue performance of a polymer modified binder measured by linear amplitude sweep test. Assessment and characterization of storage stability are thus quite important and needed for PPC modified asphalt binders, which is the main aim of this study.

The storage stability for a modified asphalt binder is generally assessed globally using the separation tube test (also called the cigar tube test), standardized as ASTM D7173 [21]. The test involves subjecting a slender aluminium tube containing modified binder sample to hot prolonged storage at 163°C for 48 h. The tube is then transferred to a freezer for 4 h at –10±10°C allowing sawing of the tube in three equal segments. The binders from the top and bottom segments are extracted and their properties are compared to evaluate the storage stability. Typically, softening points are determined for the top and bottom segment and a lower softening point difference (SPD) indicating a more storage stable binder. The analysis of differences in the top and bottom segment binders using rheological functions would be a better approach compared to SPD alone, since rheological functions such as complex modulus (G*) and phase angle (δ) are more sensitive to changes in binder properties after hot storage [19,22]. Rheology-based separation indices arrived through temperature sweep, frequency sweep, zero shear viscosity (ZSV), and multiple stress creep and recovery (MSCR) tests are used in this study for a detailed characterization of storage stability performance of PPC modified asphalt binders.

Sulfur is a very commonly used material for chemical crosslinking and improvement of storage stability in polymer and rubber modified asphalt binders. The first patents on the use of sulfur to improve the storage stability of polymer-modified asphalt binders date back to 1970s. The capability of sulfur to interact with unsaturated C = C double bonds in polymer/rubber and thus improving the storage stability has been extensively reported [23,24]. The exact mechanism of improvement in storage stability by sulfur is still not comprehensively understood. However, it is widely acknowledged that sulfur chemically crosslinks the polymer chains and connects asphalt and polymer chains through sulfide and/or polysulfide bonding, inducing improved compatibility and elasticity in the modified binders [23,25–28]. Xiao et al. [28] reported a positive effect of sulfur in inhibiting the separation in SBS/CRMA (SBS: styrene butadiene styrene; CRMA: crumb rubber modified asphalt) composite modified asphalt binders. The sulfur dosage used in the study was 0.15% (by weight of asphalt). Padhan et al. [29] used three dosages of sulfur (0.1%, 0.5% and 1% by weight of asphalt) in an attempt to improve the storage stability of EVA-polyoctenamer (EVA: ethyl vinyl acetate) modified binders. The SPD results showed that sulfur promoted the storage stability performance up to 0.5% dosage. An increase in SPD was found at 1% indicating that 0.5% was the optimum dosage. A 0.195% sulfur dosage was used in the study by tur Rasool and co-workers [30] for asphalt binders modified with truck tire rubber. It was reported that the crosslinking of sulfur with rubber particles and the formation of C-S bonds with asphalt binder prevented phase separation between asphalt and rubber. In another study, Mandal et al. [31] used seven crosslinking agents including sulfur (sulfur dosages were 0.1% and 0.225% by weight of asphalt) to evaluate the performance of binders modified with linear and radial elastomers, and functionalized polyethylene. The findings showed that the type and concentration of a crosslinking agent had a significant effect on the storage stability performance and sulfur was ranked as one of the best crosslinking agents. Review of literature thus indicates the success of sulfur toward better storage stability of modified asphalt binders.

**Fig 1:**
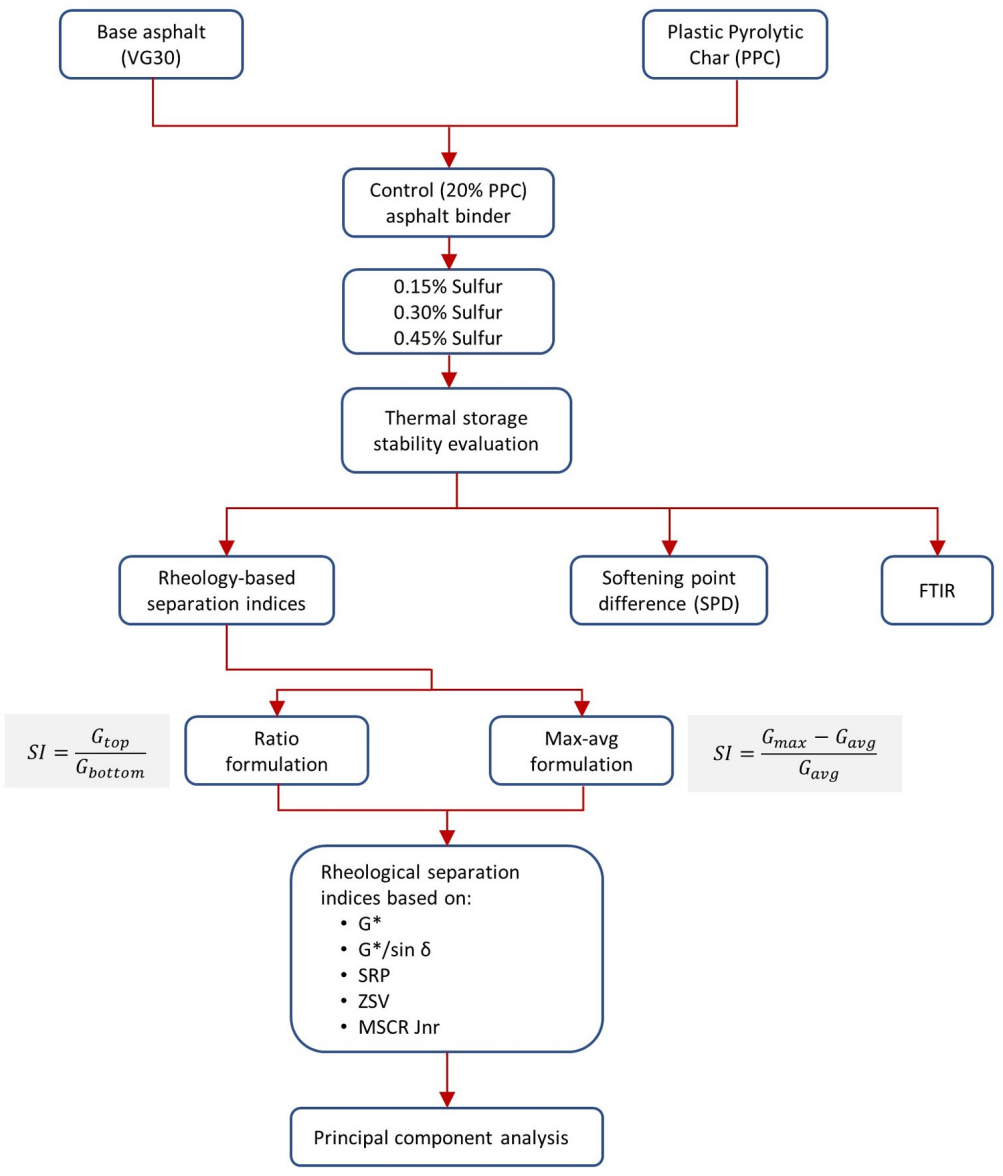
Experimental programme of the study.

A storage stable formulation of PPC modified asphalt binders will contribute to realize the positive impact of the modification and promote its wide application in pavement construction. No study is found on evaluation and characterization of thermal storage stability of PPC modified asphalt binders, which forms the novelty and need of this work. A high PPC dosage of 20% (by weight of binder) was used in this study considering that storage stable formulation with high PPC dosage would further help to promote its use in higher quantities, and such dosages have also been used previously for asphalt binder modification [12,32]. The storage separation was quantified using SPD and five rheological indices based on complex modulus (G*), Superpave rutting parameter (G*/sin δ), Shenoy rutting parameter (SRP), ZSV and MSCR unrecovered compliance (*J*_*nr*_). Two formulations of each rheology-based separation index were studied: (1) ratio, and (2) maximum-average difference formulations. Fourier transform infrared spectroscopy (FTIR) was then also used to compare the chemical functionalities of binders to judge storage stability. Principal component analysis was also performed to ascertain if the way of formulation of the separation index results in clusters with similar sensitivity. Fig 1 shows the experimental programme used in the present study.

## Materials and methodology

### Materials

**Fig 2:**
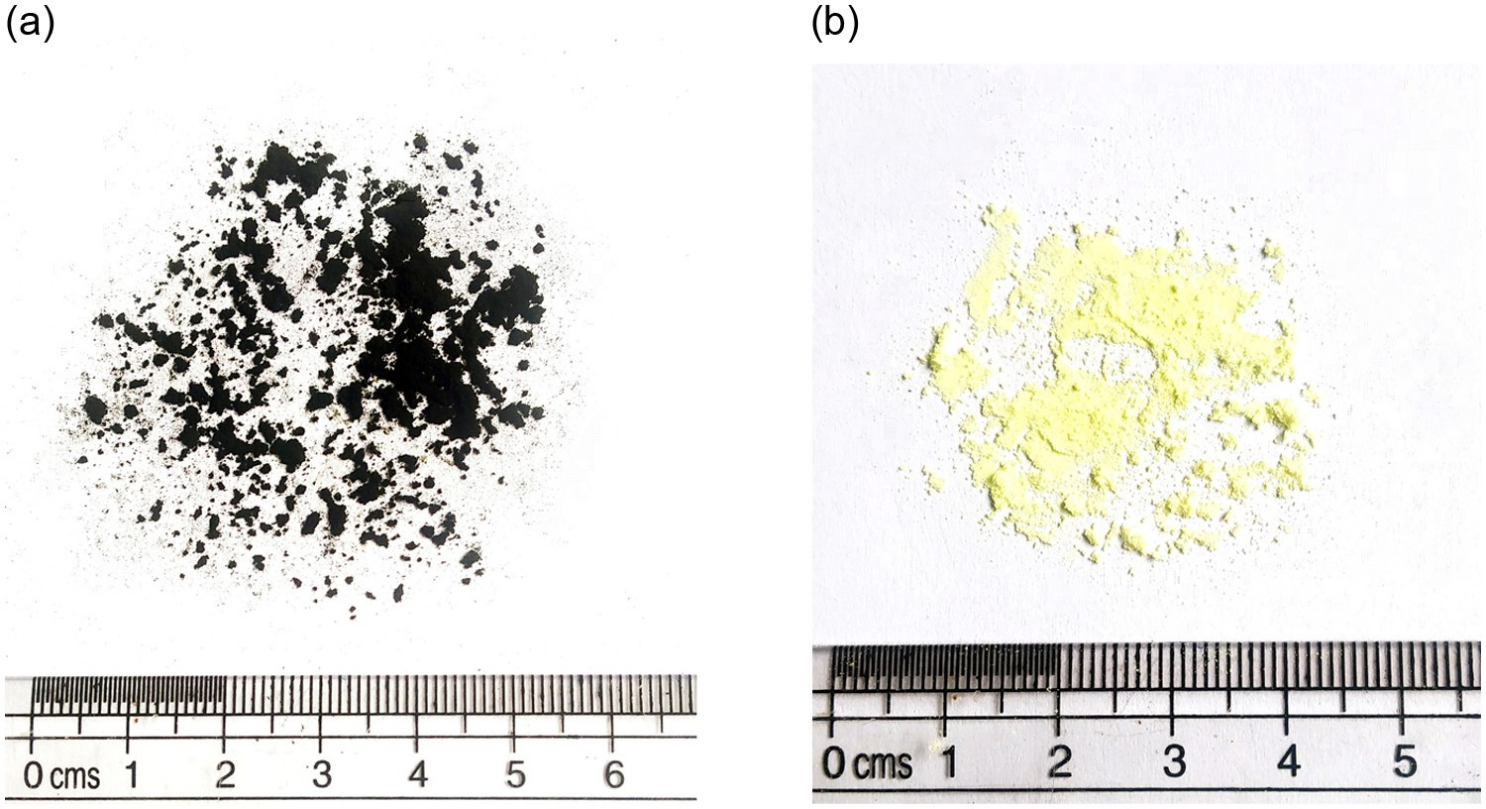
Visual appearance of (a) PPC, (b) sulfur.

A viscosity grade (VG30) unmodified asphalt binder was used in this study as the base binder for PPC modification. The main properties of the binder were: penetration 51.6 dmm; softening point 52.7°C; absolute viscosity (at 60°C) 3410 poise; kinematic viscosity (at 135°C) 525 cSt. The PPC obtained from Innova Engineering and Fabrication (Mumbai, India) was a by-product generated in industrial-scale plastic waste pyrolysis. Details of the pyrolysis process employed are described elsewhere [32]. The obtained PPC was oven-dried and PPC particles passing a 75 μm sieve were used for binder modification. Elemental sulfur powder, yellow in appearance having 99.9% purity was used as a cross-linking agent. The base binder and sulfur were supplied by TikiTar Industries (Gujarat, India). Three sulfur dosages were employed in this study (0.15%, 0.3%, and 0.45% by weight of the modified asphalt) based on the findings of the studies discussed earlier. The visual appearances of PPC and sulfur are shown in Fig 2.

**Fig 3:**
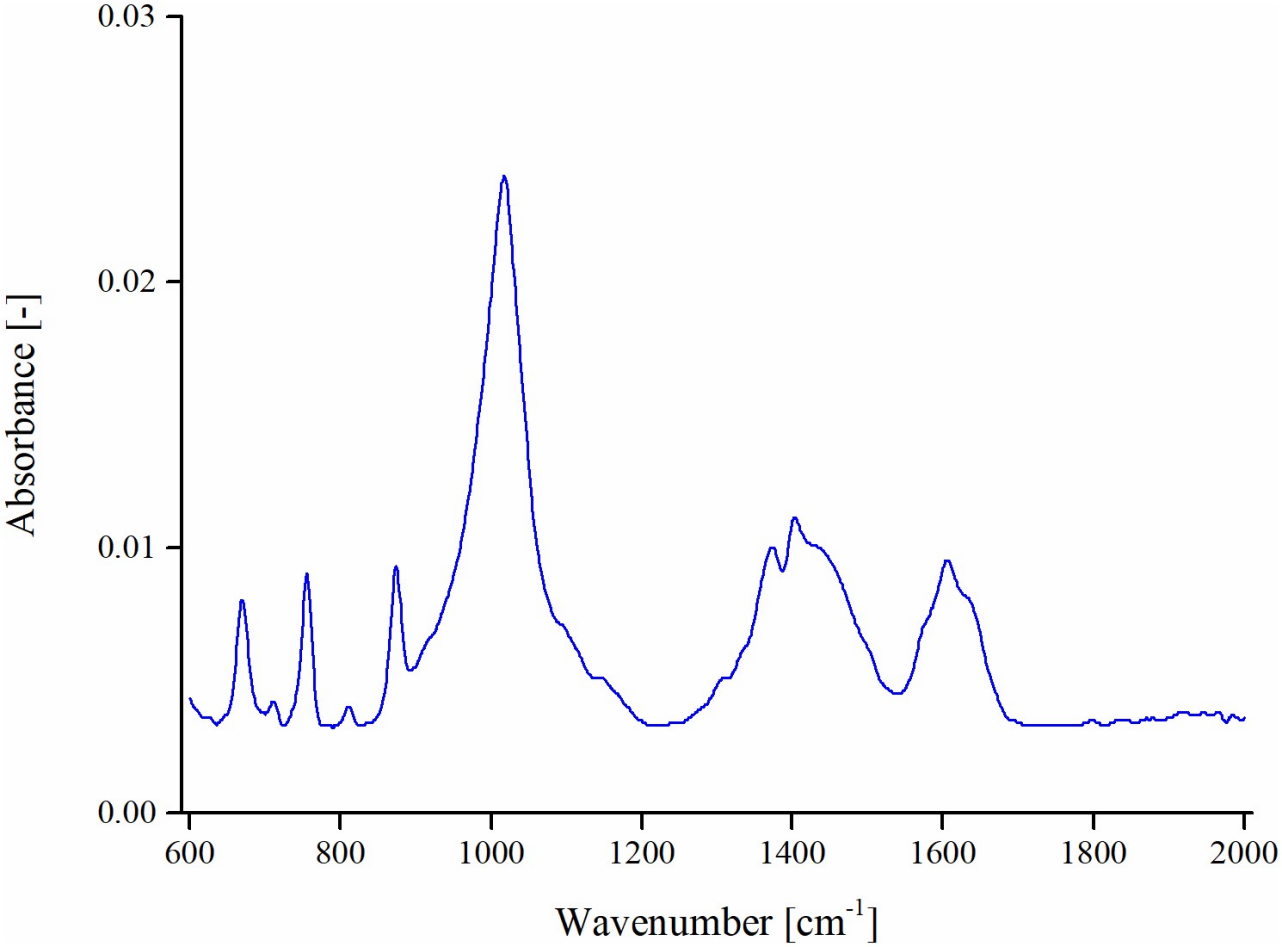
FTIR spectrum of PPC.

FTIR spectroscopy was employed for identifying the presence of chemical functional groups in PPC. The FTIR spectral curve of PPC is presented in Fig 3 for 600–2000 cm^-1^ range. The observed FTIR spectral peaks for PPC are identified based on past literature [33,34]. The spectral peak observed around 670 cm^-1^ corresponds to C = C bending of disubstituted alkene class and absorption peaks at 754 and 875 cm^-1^ belong to disubstituted C-H bending. A strong spectral band at 1020 cm^-1^ is due to aromatics of C-O stretching. The spectral bands between 1300–1410 cm^-1^ are assigned to aliphatic—C-H bending and belong to the alkane group. The absorption peak at 1610 cm^-1^ shows the presence of unsaturated ketone corresponding to C = C stretching.

### Preparation of PPC modified asphalt binder blends

PPC modified binder was prepared with 20% PPC content by weight of the neat asphalt. All binders were prepared using a high shear mixer (IKA T-25 ULTRA TURRAX) at standard designed blending parameters: blending temperature = 160°C; blending time = 30 min and shear rate = 12,000 rpm. On reaching the temperature of 160°C, the base (unmodified) binder was charged with incremental PPC amounts. To assess the effect of sulfur as a cross-linking additive in enhancing the thermal storage stability of PPC modified binders, sulfur was added at varying dosages of 0.15, 0.30 and 0.45% (by binder weight) upon the completion of blending with PPC, and the mixing was continued for an additional 30 min. The modified binder with 20% PPC dosage alone (without sulfur) is designated as ‘control’ while the binders with sulfur contents of 0.15%, 0.3%, and 0.45% are respectively designated as ‘PPC-0.15S’, ‘PPC-0.3S’, and ‘PPC-0.45S’ throughout this manuscript.

### Storage stability test

The storage stability test, also known as the cigar tube test, was carried out according to ASTM D7173 [21] utilizing a 50 g modified asphalt binder sample conditioned in an aluminium tube (diameter: 25 mm; length: 140 mm) maintaining an upright position throughout the test. After charging the tube with the sample, the tube was subjected to quiescent thermal storage at 163°C for 48 h, followed by placement of the tube at –10°C in a refrigerator for 4 h to solidify the sample. The tube was then sawed in three segments (top, middle, and bottom) and modified asphalt binder samples were extracted from the top and bottom segments discarding the middle one. The top and bottom segment samples were submitted to softening point and rheological tests. A lower softening point difference (SPD) or less difference in properties of the binders from the top and bottom sections correspond to better storage stability of the modified asphalt binder.

### Rheological tests

Three rheological tests were performed on the PPC modified asphalt binders and binder samples extracted from the top and bottom segments in the cigar tube test: temperature sweep, frequency sweep, and MSCR. An Anton Paar modular compact rheometer (MCR-102) dynamic shear rheometer (DSR) was used to perform the tests using 25 mm parallel plate geometry and 1 mm gap between the plates. In the temperature sweep test, the test temperature was varied from 40 to 80°C at 10 rad/s (1.59 Hz) frequency. Test frequencies were varied from 0.1 to 100 rad/s in the frequency sweep test at a constant high pavement temperature of 60°C. The strain amplitude was fixed at 0.1% for temperature and frequency sweep tests. Along with the two main binder linear viscoelastic parameters complex modulus (G*) and phase angle (δ), zero shear viscosity (ZSV) was used in the evaluation of PPC modified asphalt binders. ZSV is an asphalt binder rheological property used for characterization of its rutting resistance with a higher value indicating higher resistance to permanent deformation. ZSV of the modified binders was determined from the complex viscosity (η*) versus frequency data generated in the frequency sweep test. The data were fit to Cross model (Eq 1):

$$\eta *=\frac{\eta_{0}-\eta_{\infty }}{1+{\left(k\omega \right)}^{m}}+\eta_{\infty }$$ (1)

where, η* = complex viscosity (Pa.s); *η*_*0*_ = ZSV (Pa.s); *η*_*∞*_ = limiting viscosity; *ω* = angular frequency (rad/s), *k* and *m* are constants.

For further rheological characterization of rutting behavior, multiple stress creep and recovery (MSCR) test was conducted. Good correlations have been reported for MSCR test results with field rutting [35,36]. In the MSCR test, a binder sample was subjected to three stress levels (0.1, 3.2, and 10 kPa) for 30 creep-recovery cycles at each stress level. A higher level of creep recovery cycles is used to avoid variations usually observed during the conventionally used 10 cycles. The creep period was 1 s followed by 9 s recovery period. The strain was measured as a function of time. For an MSCR cycle, creep strain (*ε*_*c*_: strain at the end of the 1 s creep), unrecovered strain (*ε*_*nr*_: strain at the end of the 9 s recovery) and the recovered strain (*ε*_*r*_) was measured to obtain the unrecovered creep compliance (*J*_*nr*_) and percent recovery as per Eqs 2 and 3 respectively. Stress sensitivity of the PPC modified binders was evaluated based on the difference in *J*_*nr*_ at 0.1 and 3.2 kPa stress levels, as shown in Eq 4. The average *J*_*nr*_ and recovery data of the last five MSCR cycles were reported, as suggested in some recent studies [37,38]. Two replicates were used and average values were reported.

$$J_{nr}=\frac{\varepsilon_{nr}}{\sigma }$$ (2)

$$\%R=\frac{\varepsilon_{r}}{\varepsilon_{c}}\times 100$$ (3)

Jnr,diff=[Jnr]0.1−[Jnr]3.2[Jnr]0.1×100 (4)

where, *J*_*nr*_ = non-recoverable creep compliance (kPa^–1^); *ε*_*nr*_ = non-recovered strain at the end of a creep-recovery cycle, %R = percent recovery, *ε*_*r*_ = recovered strain, *ε*_*c*_ = strain at the end of creep period, and *σ* = stress level (kPa).

### FTIR spectroscopy

The chemical functionalities of top and bottom sections of binder samples were compared using FTIR spectroscopy. FTIR spectra were recorded with PerkinElmer UATR Two FTIR spectrometer operating in attenuated total reflectance (ATR) mode with a scanning range of 400–4000 cm^–1^. Binder samples were diluted in tetrahydrofuran (THF) at 10% w/v. A micropipette was used to drop 20 μl of the sample onto the sampling plate for acquiring the spectrum.

### Separation indices

In addition to SPD, researchers have attempted quantification of storage stability based on comparison of rheological parameters determined on the top and bottom segment binder samples of the cigar tube test [39,40]. In addition to conventional Superpave rutting parameter (G*/sin δ) used in performance grading to determine the binder permanent deformation resistance, Shenoy rutting parameter (SRP), a refinement of the Superpave rutting parameter with the expression G*/(1-(1/(tan δ sin δ)), is also used. With the term (1-(1/(tan δ sin δ)) being more sensitive to δ than sin δ, SRP is reported to maximize the effects of elastic behavior commonly observed in modified asphalt binders [41–43]. A separation index to characterize the storage stability can be formulated in two forms: firstly, in a ratio form (ratio of a rheological property of the top to bottom segment of cigar tube test, the first column of Table 1) and secondly in a maximum-average difference form (abbreviated as ‘max-avg’, the second column of Table 1). In some studies, the separation index has been reported in the ratio form [39,40], whereas others have used the max-avg form [44–46]. Comparison based on the form of the index would be helpful to understand if the method of formulation of a separation index affects the ranking of binders concerning their storage stability. Further, since G* and δ are properties heavily dependent on temperature and frequency, it is of interest to evaluate the variation of a separation index for a range of temperatures and test frequencies. In this study, we have used separation indices based on G*, G*/sin δ, SRP, ZSV and *J*_*nr*_ in both ratio and max-avg forms. The separation indices based on G*, G*/sin δ, and SRP are also further determined as functions of temperature and frequency. Table 1 enlists the equations of the separation indices used in this study in the two formulations.

**Table 1:** Formulations and expression for separation indices (SIs).

*Ratio formulation*	*Max-avg formulation*	*Remarks*
$\frac{{\left[G*\right]}_{top}}{{\left[G*\right]}_{bottom}}$	$\frac{{\left[G*\right]}_{\text{max}}-{\left[G*\right]}_{avg}}{{\left[G*\right]}_{avg}}$	SI based on complex modulus (G*)
$\frac{{\left[G*/sin\delta \right]}_{top}}{{\left[G*/sin\delta \right]}_{bottom}}$	[G*sinδ]max−[G*sinδ]avg[G*sinδ]avg	SI based on Superpave rutting parameter (G*/sin δ)
$\frac{{\left[SRP\right]}_{top}}{{\left[SRP\right]}_{bottom}}$	$\frac{{\left[SRP\right]}_{\text{max}}-{\left[SRP\right]}_{avg}}{{\left[SRP\right]}_{avg}}$	SI based on Shenoy rutting parameter G*/(1-(1/(tan δ sin δ))
$\frac{{\left[ZSV\right]}_{top}}{{\left[ZSV\right]}_{bottom}}$	$\frac{{\left[ZSV\right]}_{\text{max}}-{\left[ZSV\right]}_{avg}}{{\left[ZSV\right]}_{avg}}$	SI based on zero shear viscosity (*ZSV*)
$\frac{{\left[J_{nr}\right]}_{bottom}}{{\left[J_{nr}\right]}_{top}}$	$\frac{{\left[J_{nr}\right]}_{\text{max}}-{\left[J_{nr}\right]}_{avg}}{{\left[J_{nr}\right]}_{avg}}$	SI based on MSCR unrecovered compliance (*J*_*nr*_) at three stress levels (0.1, 3.2, 10 kPa)

## Results and discussion

### General rheology

#### Temperature sweep

**Fig 4:**
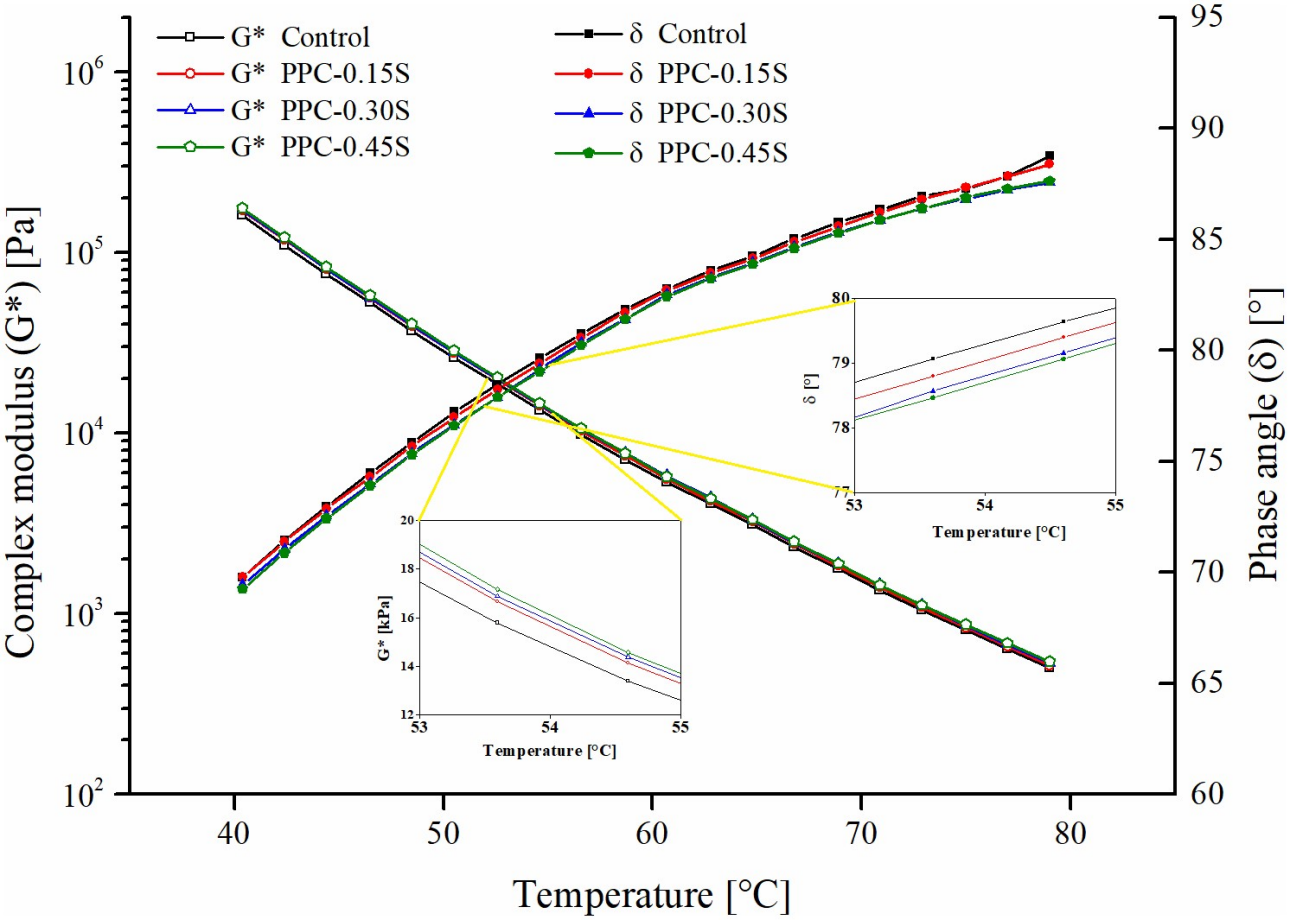
Temperature sweep results of PPC modified binders with different sulfur contents.

The complex modulus (G*) and phase angle (δ) results measured over 40 to 80°C in the temperature sweeps are shown in Fig 4. With an increase in temperature, there is a decrease in G* and increase in δ for all asphalt binders, which is expected as a higher temperature reduces the binder stiffness and elasticity. With regard to the effect of sulfur, there is a slight increase in G* and a slight decrease in δ at all temperatures after adding sulfur. This indicates that there is a small improvement in the deformation resistance and elastic behavior of PPC modified asphalt with the addition of sulfur. Averaged over the entire range of temperatures, sulfur increases G* by 4%, 7% and 8% respectively at 0.15%, 0.3% and 0.45% dosages compared to the control. It is also observed that the increase in G* and decrease in δ persists over the entire temperature range. The slopes of the logarithm of G* with temperature for all binders were found very close to each other, suggesting that the addition of sulfur did not change the temperature susceptibility of the PPC modified binder.

### Frequency sweep

**Fig 5:**
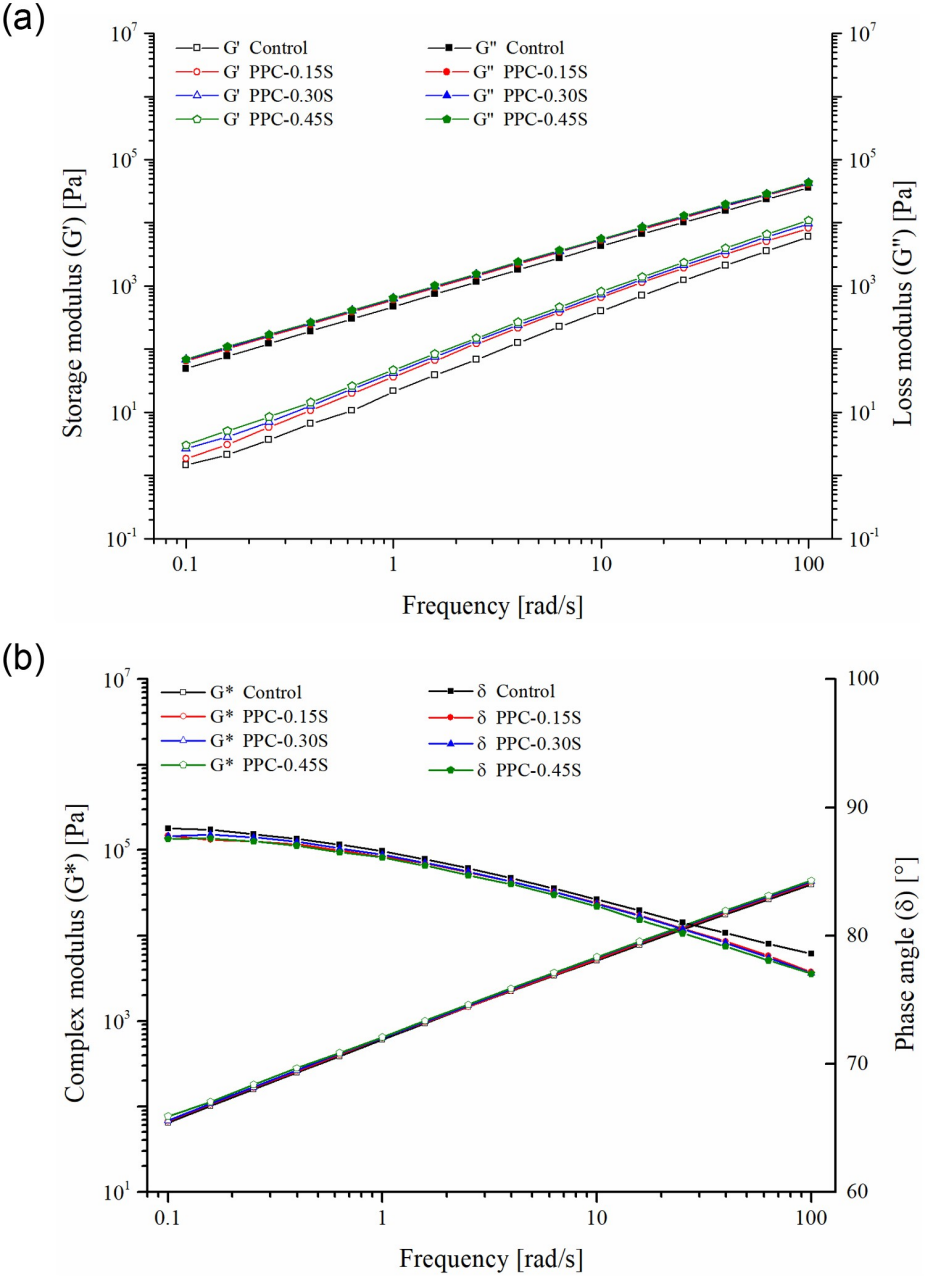
Frequency sweep results (a) storage and loss moduli (b) G* and δ.

**Fig 6:**
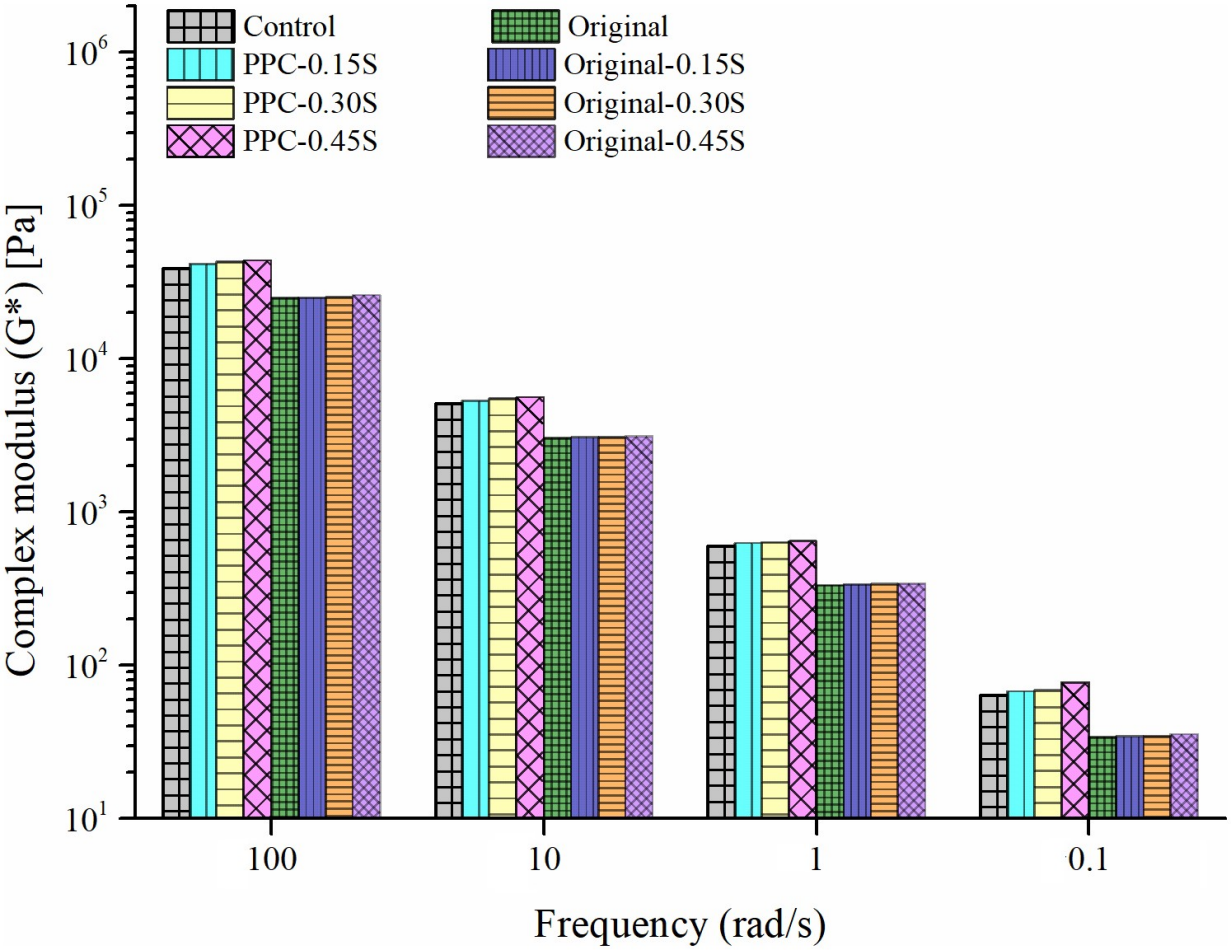
G* for unmodified and PPC modified binders with sulfur at multiple frequencies.

The dependence of storage (G′) and loss moduli (G″) on loading frequencies at 60°C temperature is shown in Fig 5a. A 60°C temperature was selected since it is generally used to evaluate the binder performance at high pavement service temperatures. Sulfur causes small improvement in the storage modulus of the binders and higher improvements are observed at higher dosages. Similarly, Fig 5b shows a slight increase in G* and a slight decrease in δ values with the increase in sulfur content. Zhang et al. [26] observed degradation of the crosslinked polymer network at frequencies higher than 2.5 rad/s that led to a decrease in G* for SBR modified binders with sulfur. The authors noted that the vulcanized binder was more susceptible to dynamic shear loading (at higher frequencies). However, in this study, the increase in G* and decrease in δ (shown in Fig 5b) persist over the entire frequency range (0.1 to 100 rad/s), which also agrees with the results of temperature sweep tests. This finding suggests that the network formed in the PPC modified binder on the addition of sulfur is able to persist throughout the range of temperatures and frequencies chosen. To further investigate the interaction of sulfur with PPC modifier, sulfur-asphalt blends without PPC were prepared at the same sulfur dosages with the unmodified (base) VG30 binder. The G* of the unmodified and the PPC modified binders at selected frequencies with multiple sulfur dosages are presented in Fig 6. It is seen that the effect of sulfur is present in PPC modified binders whereas almost constant G* values are obtained for the unmodified binder at multiple sulfur contents. Therefore, sulfur interacts with PPC modified binders and the interaction has a positive effect, no matter small, on the rheological performance.

**Fig 7:**
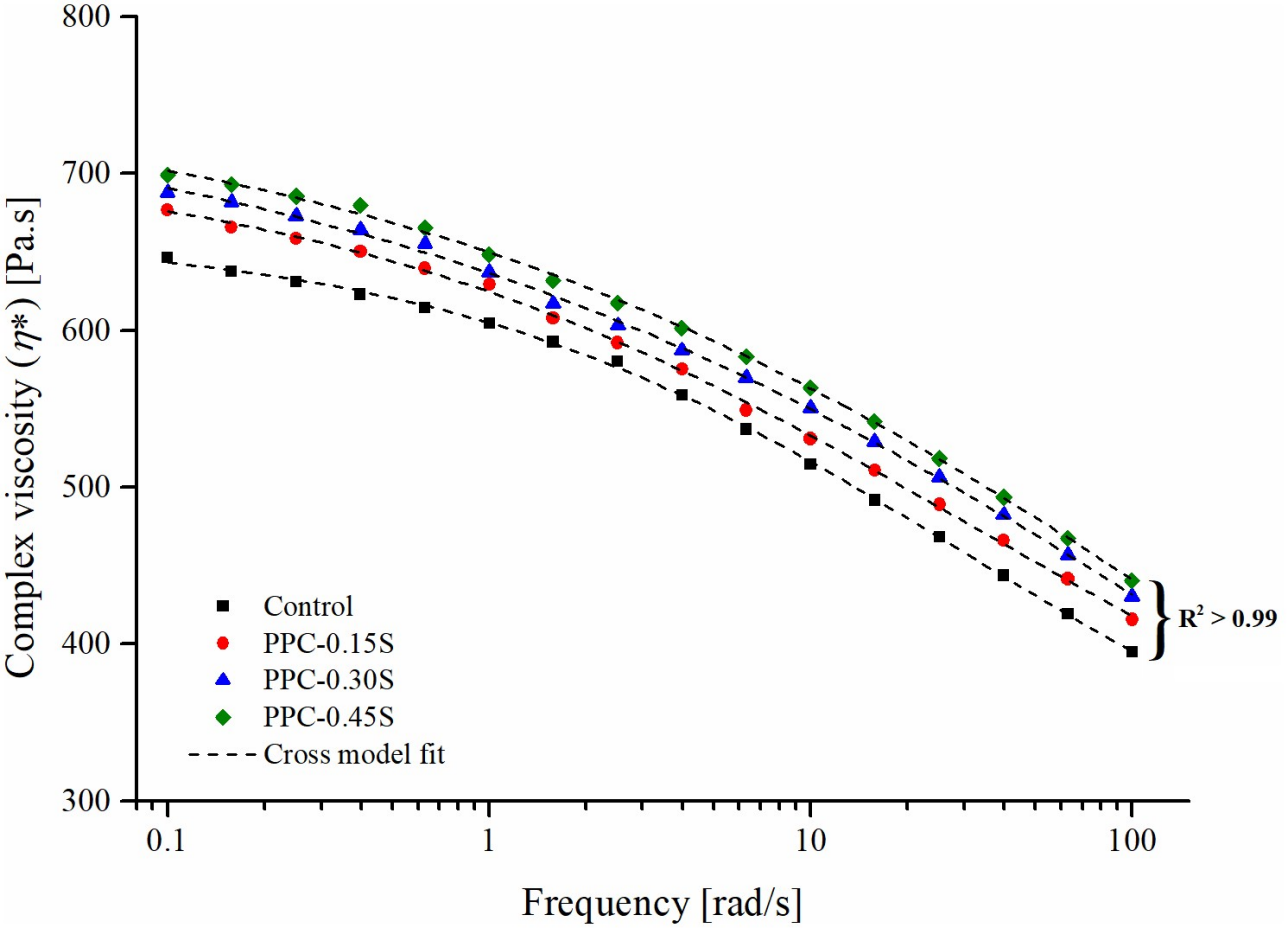
Cross model fit to complex viscosity-frequency data for PPC modified binders.

**Fig 8:**
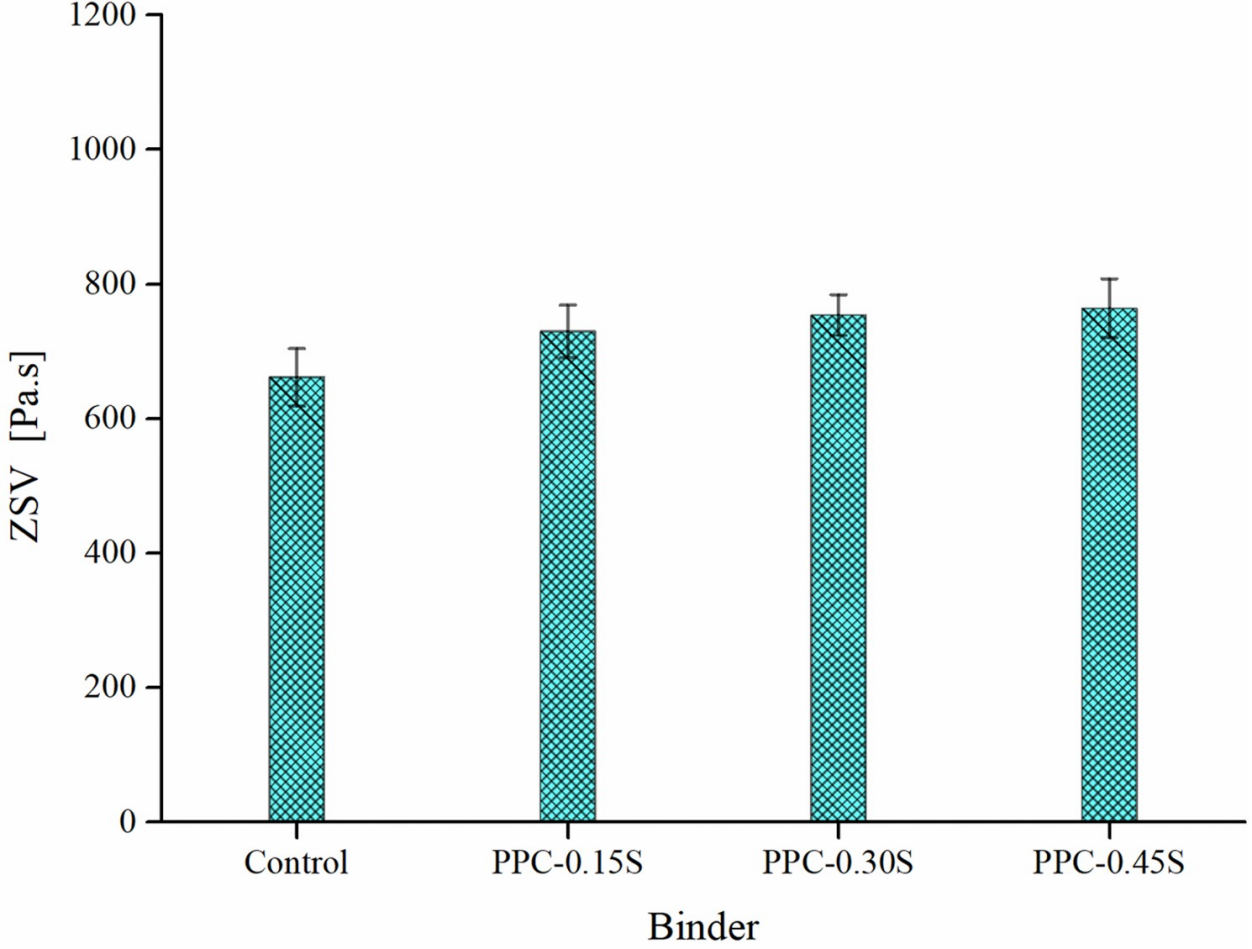
ZSV results.

The zero shear viscosity (ZSV) of the PPC modified binders was determined at 60°C based on Cross model fit of complex viscosity (η*) versus frequency data. Since modified asphalt binders typically exhibit non-Newtonian behavior (viscosity not independent of shear rate/frequency), the ZSV is determined as a steady-state viscosity at very low shear conditions maintaining equilibrium without causing structural changes in the binder [47,48]. Fig 7 shows the Cross fit to the η*-frequency data for the PPC modified binders at different sulfur contents. A good fit can be seen in all cases with R^2^ ≈ 0.99. ZSV is used as a parameter reflecting the permanent deformation performance of the binder. As shown in Fig 8, the trend of ZSV is similar to that of other rheological variables with a small increase at increasing sulfur dosages, indicating that the permanent deformation behavior of PPC modified binders is slightly improved by the addition of sulfur.

#### MSCR

**Fig 9:**
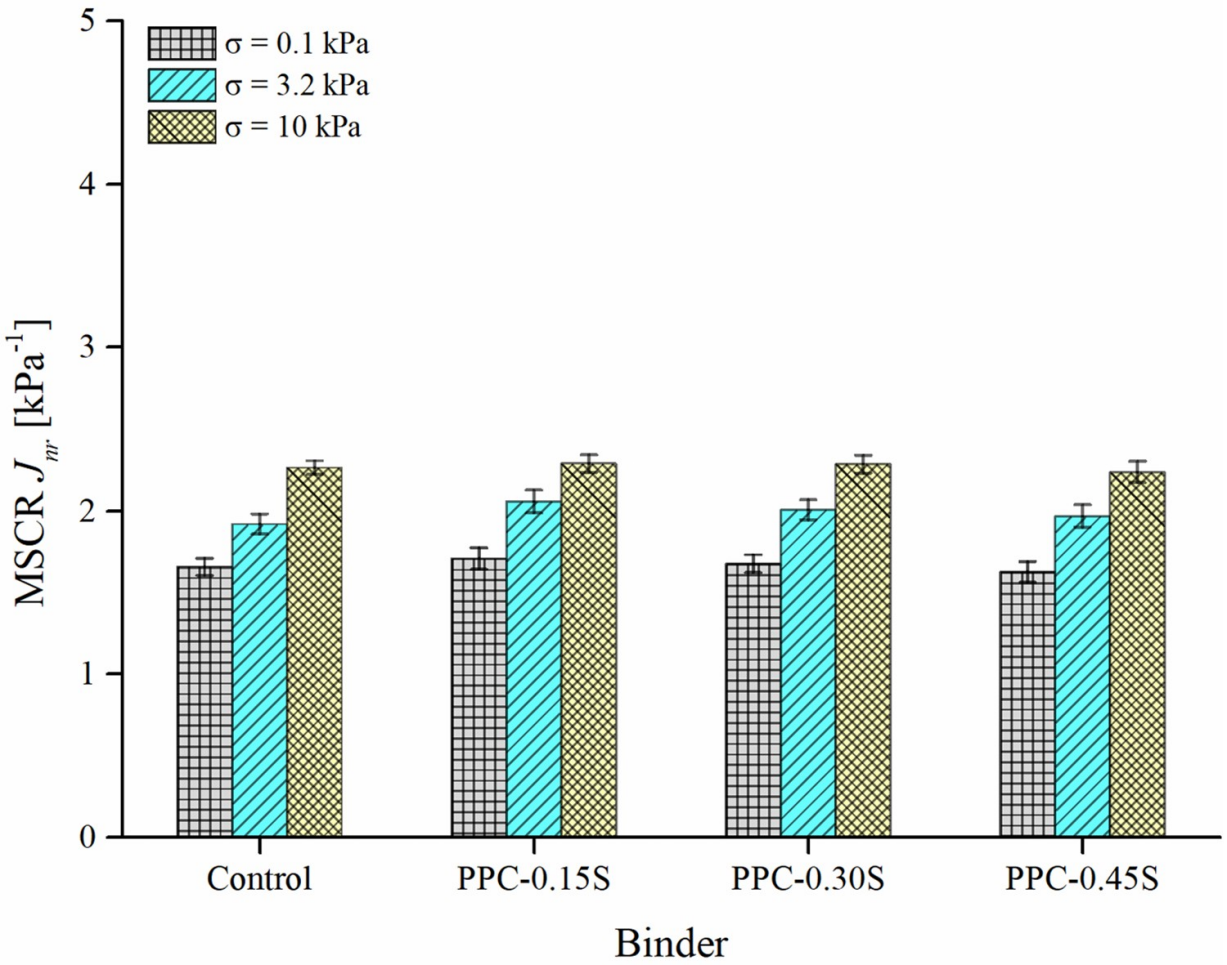
MSCR *J*_*nr*_ results.

**Fig 10:**
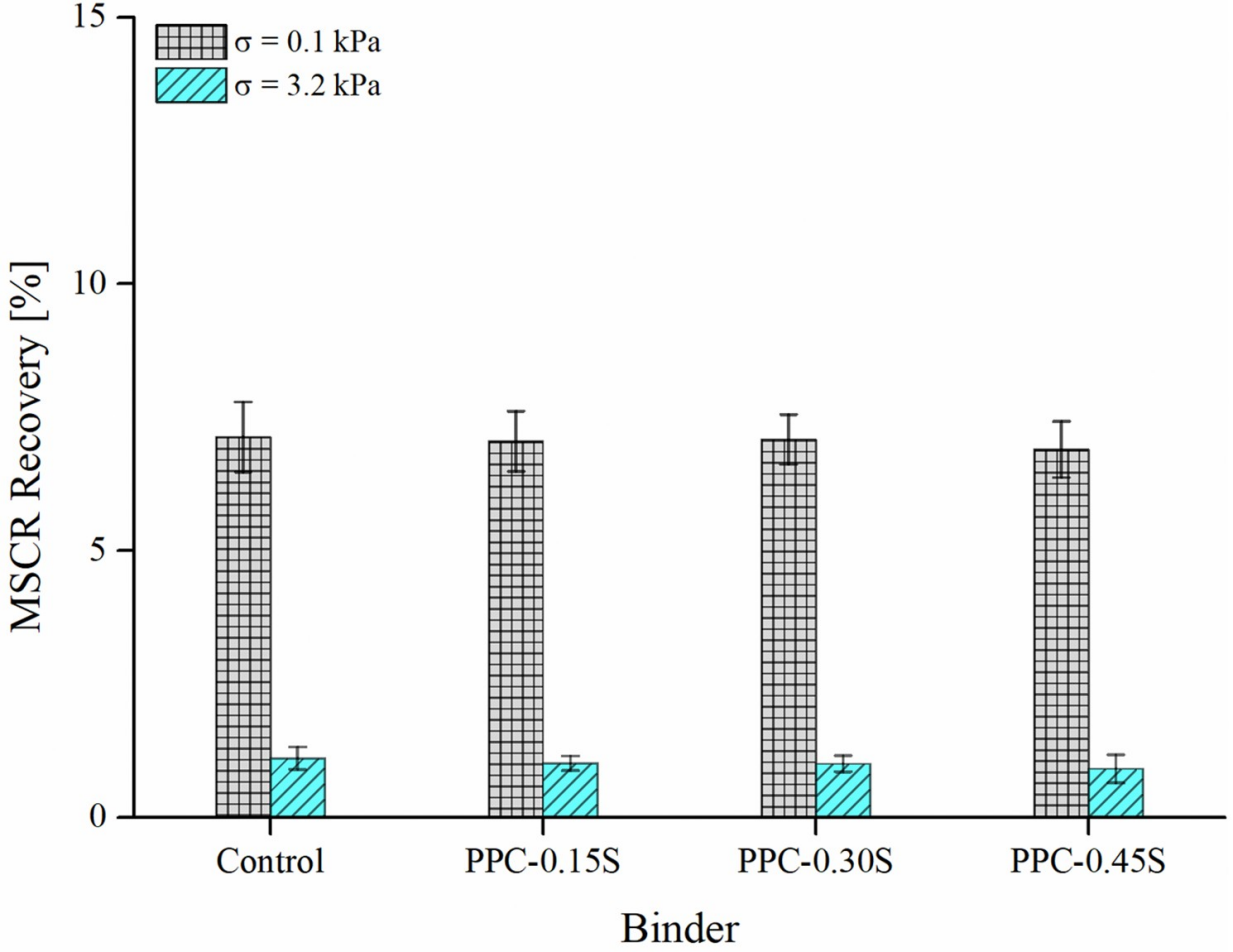
MSCR recovery results.

Figs 9 and 10 respectively present the results of MSCR *J*_*nr*_ and percent recovery of PPC modified binders at various sulfur contents. The test was performed at 60°C. The *J*_*nr*_ is a measure of the strain unrecovered at the end of the recovery period in MSCR creep-recovery cycles normalized to the stress applied, and therefore a lower value is desirable. Similarly, a higher recovery is desirable for binder related rutting performance as it indicates a higher ability of the binder to restore the creep strains. The results from Fig 9 indicate that the addition of sulfur has little to no effect on the MSCR *J*_*nr*_. A similar observation can also be made for percent recovery at both stress levels (0.1 and 3.2 kPa) from Fig 10. Negative recovery values were observed at 10 kPa stress level and following the recommendations of ASTM D7405 [49], the negative recoveries were regarded as zero recoveries. Possible reasons for negative recovery have been reported as instrument inertia and tertiary creep behavior commonly observed at very high stress levels [36]. Further, the stress sensitivity of binders was measured using the difference between *J*_*nr*_ at 3.2 and 0.1 kPa. A *J*_*nr*,*diff*_ value less than 75% is generally prescribed by MSCR standard specifications to ensure that the binder is not highly stress-sensitive in the nonlinear domain. For control and PPC modified binders with all sulfur dosages, the parameter *J*_*nr*,*diff*_ was found below 25%.

Although previous studies reported the profound effect of sulfur in improving the rheological properties of binders modified with polymers/rubber [23,24,29,30]; however, in this study, the findings with multiple sulfur dosages showed slight improvements to almost no change in the rheological parameters (G*, δ, G′, G″, ZSV, MSCR *J*_*nr*_, and recovery). The chemical nature of polymers and rubber is much different than that of PPC and hence the interactions of PPC with sulfur are expected to be different than the interactions with polymer/rubber. Nevertheless, the effect of sulfur was still to slightly enhance the stiffness and elastic nature of the PPC modified binders. As will be discussed in the following sections of the paper, sulfur had a profound effect in improving the storage stability performance of the PPC modified binders.

### Storage stability analysis

**Fig 11:**
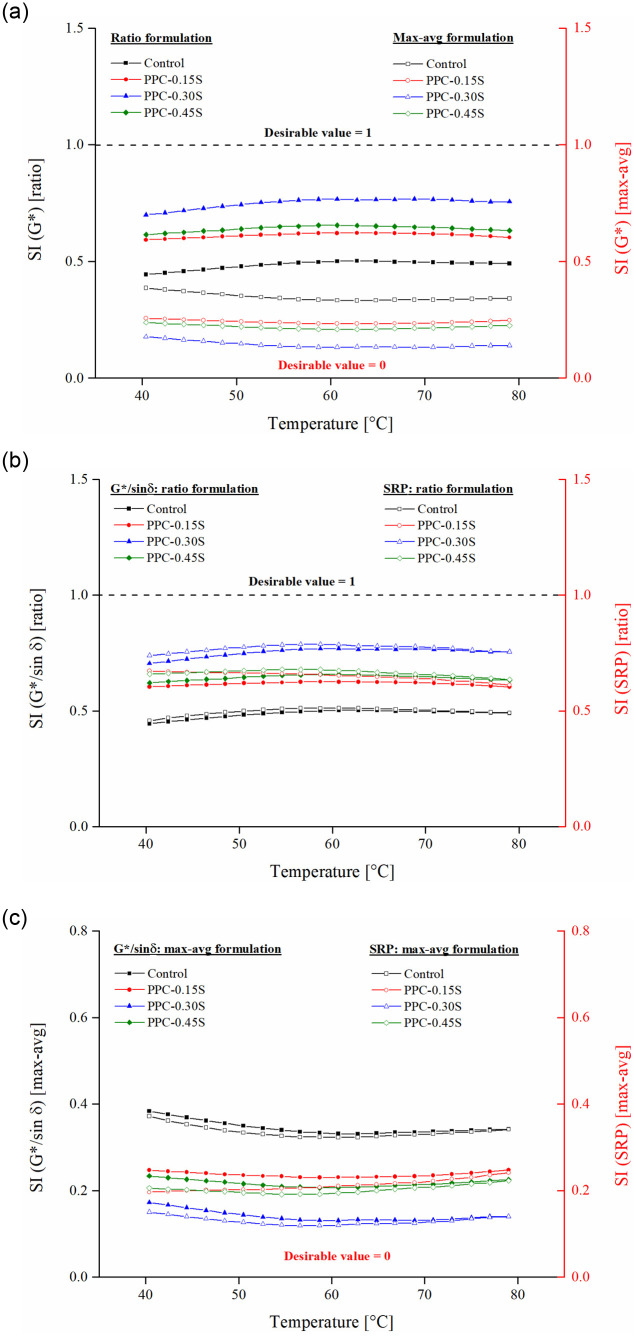
SIs as function of temperature, SI based on (a) ratio and max-avg formulations of G*, (b) ratio formulation of G*/sin δ and SRP, and (c) max-avg formulation of G*/sin δ and SRP.

**Fig 12:**
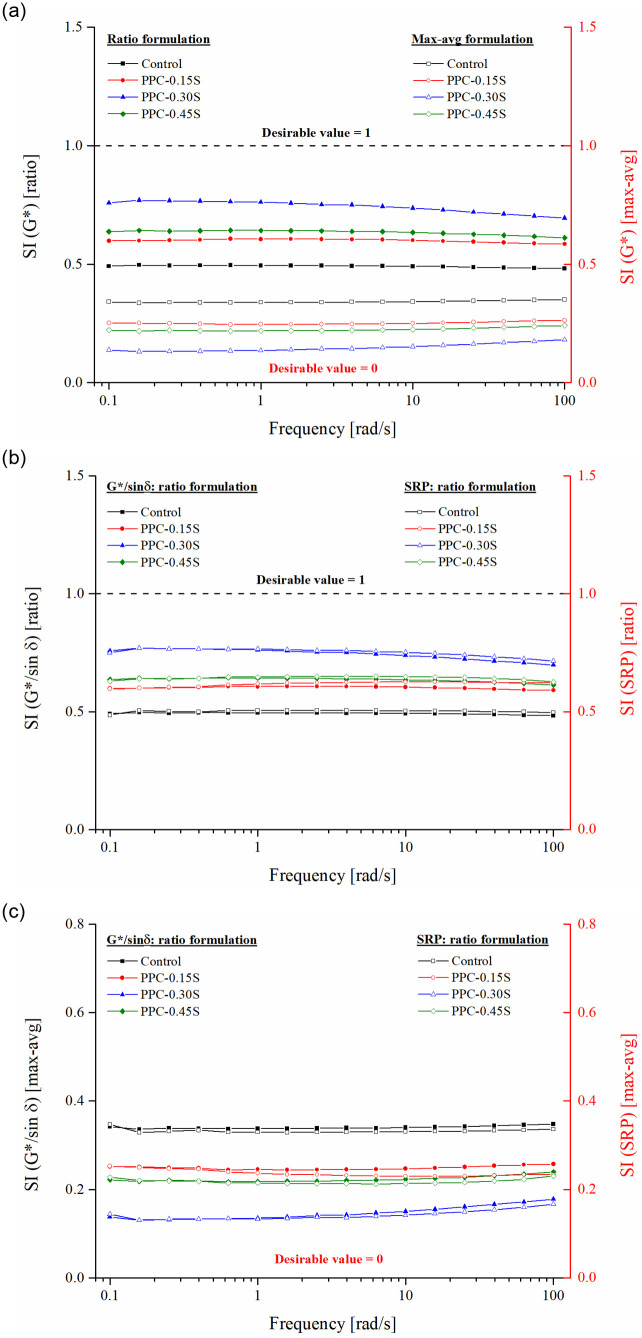
SIs as function of frequency, SI based on (a) ratio and max-avg formulations of G*, (b) ratio formulation of G*/sin δ and SRP, and (c) max-avg formulation of G*/sin δ and SRP.

SIs based on G*, G*/sin δ, and Shenoy rutting parameter (SRP) were used to characterize storage stability of PPC modified asphalt binders at different sulfur contents. SIs are presented as a function of temperatures from 40 to 80°C (Fig 11) and frequencies from 0.1 to 100 rad/s (Fig 12) under both ratio and max-avg formulations. It is to be noted that values near 1 are desirable for SIs formulated in the ratio form, whereas a lower value (near 0) is desirable for SIs formulated in the max-avg form (also indicated in the figures). The SI values become steady with fewer changes from temperatures of 55°C to 80°C. This observation is found true for both ratio and max-avg forms of the indices. A crossover of SRP SI is observed for PPC-0.15S and PPC-0.45S binders near 46°C, but beyond that, the trend of SRP SI with temperature is similar as for other indices. The variation of SIs with frequency is relatively constant except some variations observed at frequencies lower than 1 rad/s (Fig 12). The results show that the separation indices remain somewhat constant in the temperature range of 55 to 80°C and frequency range of 1 to 100 rad/s, and therefore the use of any temperature or frequency within these ranges is expected to yield the same ranking of binder separation performance.

The control PPC modified binder shows poor storage stability due to the migration of PPC particles toward the bottom of the tube during hot storage. The migration is driven by gravitational forces and can be attributed to the density difference between PPC (density = 1.74 g/cm^3^) and the base asphalt binder (density = 1.03 g/cm^3^). However, in addition to gravity, the separation may also be influenced by the composition of the base binder, PPC dosage, and PPC-asphalt chemistry. Addition of sulfur is found to improve the storage stability of the PPC modified binders as seen from the increase in ratio-based indices and decrease in max-avg-based indices. The PPC-0.3S binder with 0.3% sulfur shows the best results considering all indices in both formulations. Despite the addition of a high PPC content (20% by binder weight), the storage stability shows an appreciable improvement with the addition of sulfur. Evidently, sulfur is able to reduce the migration of PPC particles during hot storage and thus reduce the differences observed in rheological properties of the binders obtained from the top and bottom segments of the cigar tube test. It is also seen that both ratio and max-avg formulations of the indices are able to capture the differences in storage stability performance of the PPC modified binders at different sulfur dosages.

The ability of sulfur to improve storage stability in polymer/rubber modified asphalt binders has been primarily attributed to the reaction of sulfur with unsaturated C = C double bonds and the formation of interconnections crosslinking the polymer chains with themselves and with the asphalt [23,24]. The FTIR spectroscopy of PPC showed diverse chemical functional groups including the C = C double bond (Fig 3). Therefore, it is hypothesized that sulfur interacted with some PPC chemical functionalities that contributed to lower differences in the properties of top and bottom segments and thus produced a more stable binder. This hypothesis is only suggested by the improved storage stability performance and a further detailed investigation focused on the chemistry of sulfur in PPC modified asphalt binders will be helpful to identify precise chemical mechanisms contributing to the enhanced thermal separation performance.

The SI results were submitted to one-way analysis of variance (ANOVA) conducted separately on temperature and frequency sweep test results obtained in ratio and max-avg forms at a 5% level of significance. Sulfur dosage was the independent variable and the indices were response variables. Table 2 presents the multiple comparison results based on Tukey’s honest significance difference. SI based on G*/sin δ and G*–whether in ratio or max-avg forms–are found to be statistically similar. Comparing the G*/sin δ and SRP based indices, in both ratio and max-avg forms, the SRP index yields slightly higher performance in terms of separation index than the G*/sin δ index, especially at lower temperatures and higher frequencies (Figs 11b, 11c, 12b and 12c). This is attributed to the enhanced effect of the phase angle as the binder becomes increasingly elastic at lower temperatures and higher frequencies. Correspondingly, the statistical analysis reports significant differences between SRP and other indices. At higher temperatures and lower frequencies, the differences between SRP and G*/sin δ based separation indices consistently reduce.

**Table 2:** Multiple comparison results from ANOVA.

*Comparison between SIs based on*	*Formulation*	*TS data*		*FS data*	
		*p-value*	*S/NS*	*p-value*	*S/NS*
G* and G*/sin δ	Ratio	0.118	NS	0.675	NS
G* and SRP	Ratio	<0.001	S	<0.001	S
G*/sin δ and SRP	Ratio	<0.001	S	<0.001	S
G* and G*/sin δ	Max-avg	0.125	NS	0.612	NS
G* and SRP	Max-avg	<0.001	S	<0.001	S
G*/sin δ and SRP	Max-avg	<0.001	S	<0.001	S

Note: TS: temperature sweep; FS: frequency sweep; S: significant; NS: not significant.

**Fig 13:**
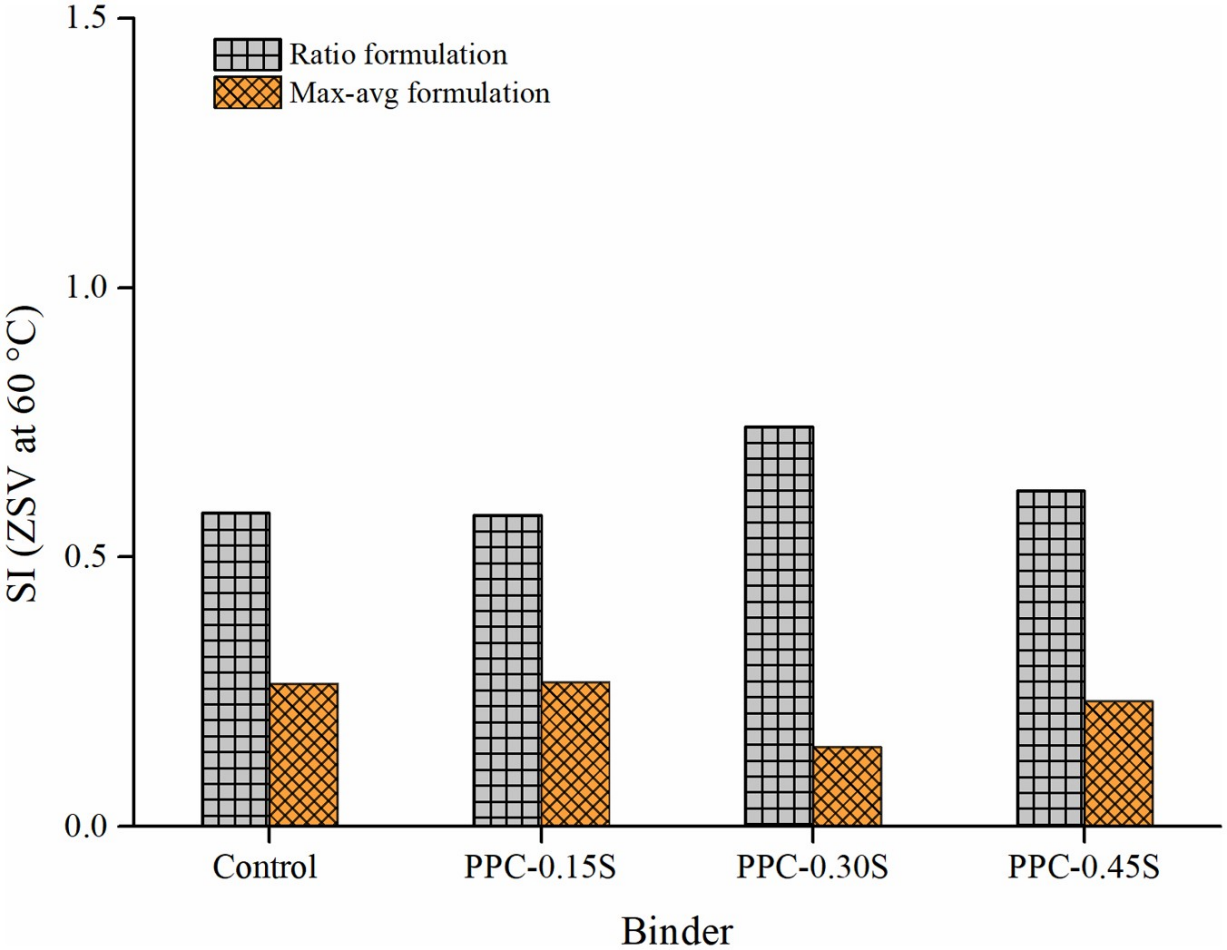
SI based on ZSV.

**Fig 14:**
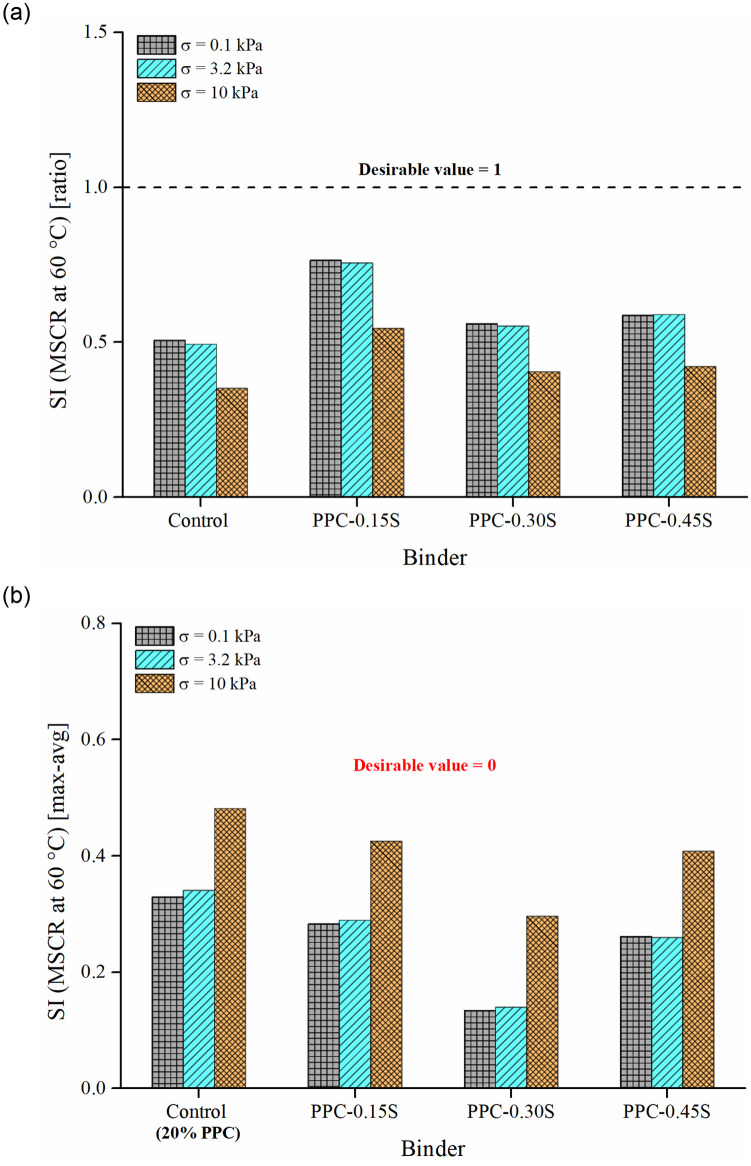
SI based on MSCR *J*_*nr*_.

Figs 13 and 14 respectively present the SIs derived from ZSV and MSCR *J*_*nr*_ in both ratio and max-avg forms. The PPC modified binder with 0.3% sulfur content again shows the least separation differences among all binders. The binders can be ranked as follows: PPC-0.3S > PPC-0.45S > PPC-0.15S> control (in the high to low order of separation performance). The observations from ZSV and *J*_*nr*_ based indices agree with those from G*, G*/sin δ and SRP based indices. According to the results shown in Fig 14, the separation indices determined by *J*_*nr*_ at 0.1 and 3.2 kPa stress levels show negligible variations compared to that shown at 10 kPa stress level. An increase in stress level from 3.2 kPa to 10 kPa in the MSCR test caused a higher increase in *J*_*nr*_ of the top segment binders than the bottom ones, therefore increasing the *J*_*nr*_ differences between the top and bottom sections. The very high stress level of 10 kPa has been recommended in the MSCR test based on the stress levels experienced by the binder in the asphalt mixtures determined based on micromechanical computations [50]. It seems that such a high stress level might not be appropriate for examining the storage stability. The usual stress levels of 0.1 and 3.2 kPa are therefore suggested as they are found to provide similar separation indices in both ratio and max-avg formulations.

**Fig 15:**
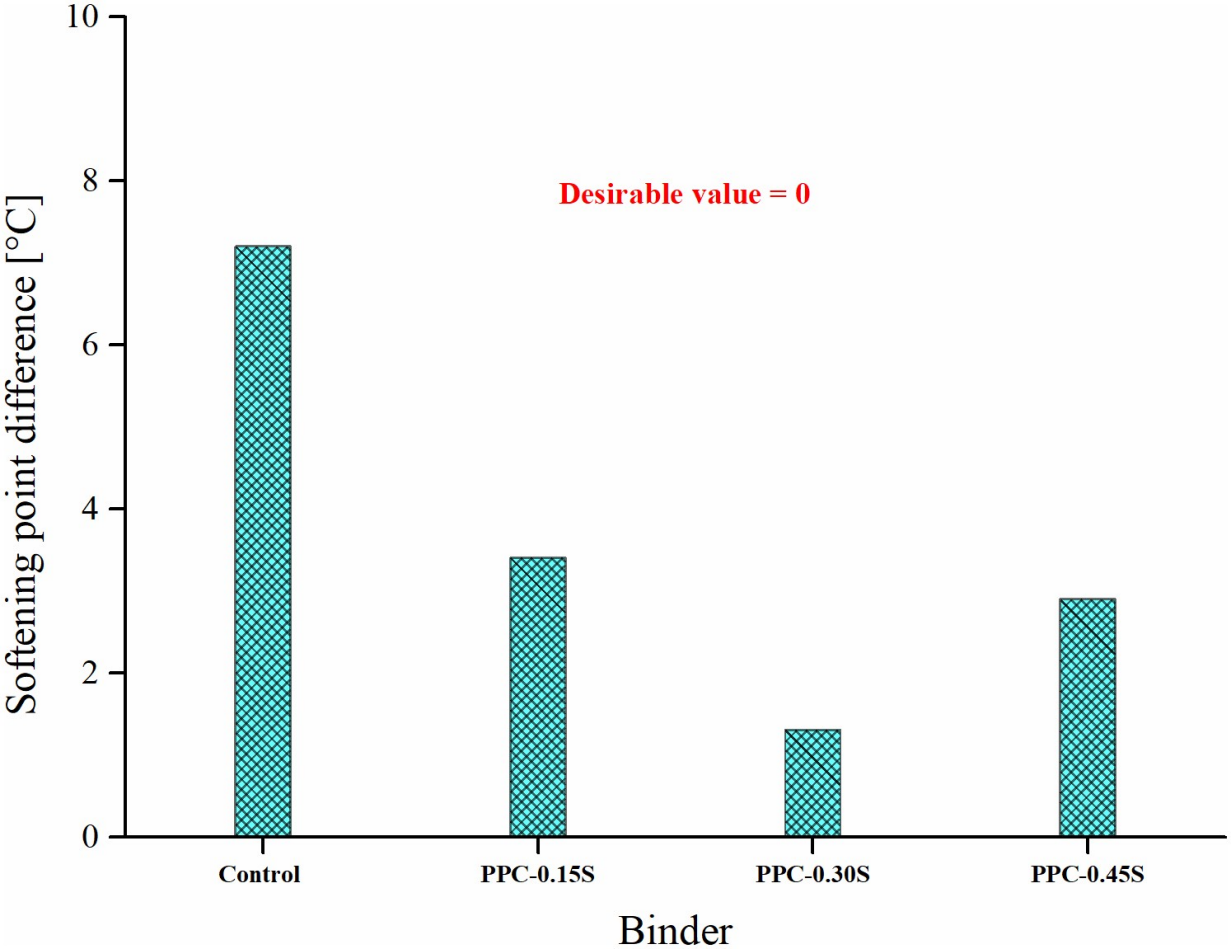
Softening point difference results.

Fig 15 presents the SPD results of the binders. The current Indian specifications for polymer [51] and rubber modified [52] asphalt binders stipulate maximum SPD values for judging the storage stability performance. Maximum SPD of 3 and 4°C have been stipulated for polymer and rubber modified binders, respectively. Notably, these specifications are not directly applicable to PPC modified binders due to obvious differences in the nature of the modifiers. However, PPC modified binders with all sulfur dosages meet the criterion for rubber modified binders as SPDs are below 4°C, whereas the binders with 0.3% and 0.45% sulfur also meet the criterion for polymer modified binders as SPD < 3°C.

**Fig 16:**
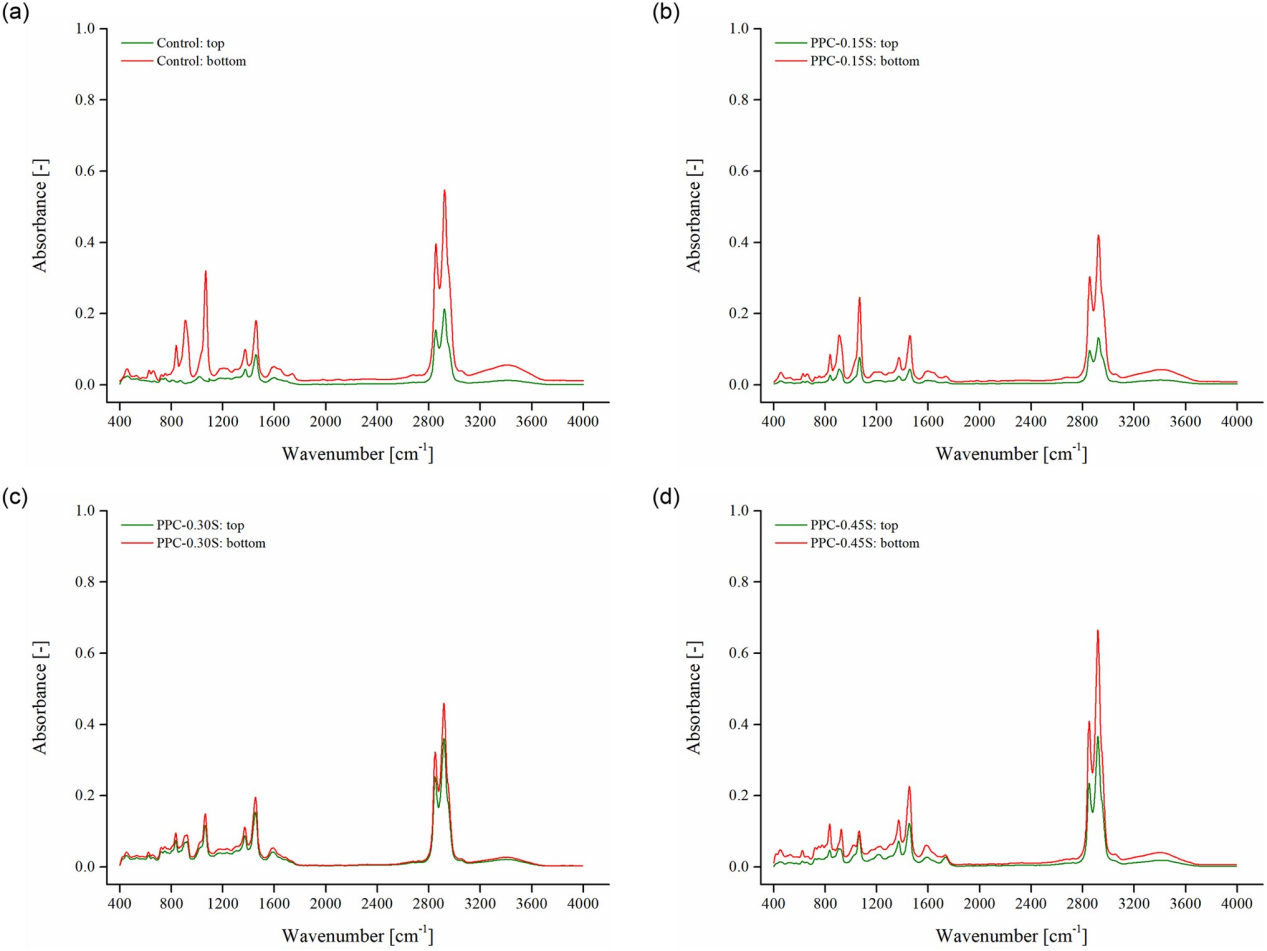
FTIR spectra of top and bottom segment binders (a) control, (b) PPC-0.15S, (c) PPC-0.3S, and (d) PPC-0.45S.

FTIR was conducted for the top and bottom sections of control, PPC-0.15S, PPC-0.30S, and PPC-0.45S samples. Fig 16 shows that major absorbance peaks occur at the same locations for the studied binders. The corresponding functional groups at the observed absorbance peaks are identified based on the existing literature [34,53,54]. The strong absorbance bands between 2800–3000 cm^–1^ exhibit the presence of aliphatic—C-H stretching of alkane compound class. The spectral peak at about 1620 cm^–1^ corresponds to C = C stretching and belongs to unsaturated ketone. The absorptions peaks around 1465 cm^–1^ and 1380 cm^–1^ are assigned to—C-H bending and affiliated to aldehyde and alkane classes, respectively. The spectral peak at 1070 cm^–1^ is attributed to S = O stretching. Additionally, the absorbance peaks between 905–915 cm^–1^ and 790–840 cm^–1^ suggest the presence of C = C bending and belong to monosubstituted and trisubstituted alkene, respectively. Since new chemical functionalities are not identified in the FTIR, a qualitative analysis is performed by inspecting the differences in the overall FTIR spectra between the top and bottom segment binders. It can be seen in Fig 16 that the spectral curve for the bottom segment of the control binder has a higher absorbance peak than the top segment suggesting that the bottom part has a higher concentration of PPC particles. A similar observation was reported by Lu et al. [55] with higher absorbance peaks in the spectra of the top segment of SBS-modified binder than the bottom segment, which was ascribed to the presence of higher SBS concentration in the top section. Fig 16 further shows that the incorporation of sulfur enhances the homogeneity as the spectral curve for top and bottom sections come closer and 0.3% sulfur exhibits the least differences. A similar conclusion was also made by Zhang et al. [56] where the FTIR spectra of the top and bottom sections of a bio-asphalt binder were in close proximity after thermal storage. The observed trends for FTIR agree with the rheological separation indices with the PPC-0.30S binder showing the best storage stability performance.

### Principal component analysis

The central idea of principal component analysis (PCA) is dimensionality reduction in datasets with a fairly large number of interrelated variables by a linear transformation of the original variables to ‘principal components (PC)’ such that the first few PCs explain most of the variation present in the original variables [57]. The PCs are orthogonal to each other (having no correlation among themselves) and are computed from the solutions of eigenvalue-eigenvector problems using matrix algebra. PCs are ordered such that PC1 (the first principal component) accounts for more data variation than PC2 (the second principal component) and so on [58]. The principal component approach has been well applied in asphalt related studies to detect patterns in the data and identify similar clusters of data [59–62].

In the present study, principal component analyses were performed on six features (three SIs based on G*, G*/sin δ, and SRP in both ratio and max-avg forms) separately on the data from temperature sweep and frequency sweep tests. The objective of the analysis was to ascertain whether the way of formulation of the separation index results in similar clusters with similar sensitivity. Other indices (ZSV, *J*_*nr*_) were not considered for PCA as they were calculated at a single temperature while G*, G*/sin δ and SRP based indices provided sufficient values with respect to temperature and frequency. The variables were centred and scaled since the original variables were on different measurement scales. The results of the analyses yielded six principal components as shown in Table 3. Each principal component is a normalized linear combination of the original six variables. The first principal component (PC1) alone accounted for more than 99% variability in the data, implying that the six variables could be transformed to a single virtual variable that accounts for majority of the variability in the original data. Other five principal components did not have an important role in terms of variability. Being the most important principal component, only the PC1 is discussed further.

**Table 3:** Principal components obtained from PCA.

*Attribute*	*PC1*	*PC2*	*PC3*	*PC4*	*PC5*	*PC6*
	*PCA on temperature sweep data*					
Standard deviation	2.4416	0.1849	0.0656	0.0059	0.0040	0.0002
Proportion of variance	0.9936	0.0057	0.0007	0.0000	0.0000	0.0000
Cumulative proportion	0.9936	0.9993	0.9999	1.0000	1.0000	1.0000
	*PCA on frequency sweep data*					
Standard deviation	2.4468	0.1009	0.0538	0.0035	0.0027	0.0001
Proportion of variance	0.9978	0.0017	0.0005	0.0000	0.0000	0.0000
Cumulative proportion	0.9978	0.9995	1.0000	1.0000	1.0000	1.0000

Table 4 shows rotations or loadings that are the coefficients of the linear combination of the original variables contributing to the PC1. Loading values lie between negative 1 and positive 1. It is seen that the loadings for ratio formulated indices are positive while those for max-avg formulated indices are negative. Therefore, ratio formulated indices have a positive relationship with PC1 (as the values of the indices increase, the PC1 also increases). On the other hand, max-avg formulated indices have an inverse relationship with PC1 (as the values of the indices increase, the PC1 decreases). More importantly, all the six indices have about the same contribution to PC1, indicating that they have a lot in common and about the same sensitivity such that they can be translated to a single principal component. It can be concluded that both ratio and max-avg formulations of separation indices are equally capable of characterizing the storage stability characteristics of PPC modified asphalt binders at different sulfur contents.

**Table 4:** PCA loadings for the first principal component (PC1).

*SI based on*	*Formulation*	*TS*	*FS*
G*	Ratio	0.4081	0.4081
G*	Max-avg	–0.4088	–0.4085
SRP	Ratio	0.4076	0.4082
SRP	Max-avg	–0.4067	–0.4077
G*/sin δ	Ratio	0.4089	0.4084
G*/sin δ	Max-avg	–0.4093	–0.4086

Note: TS: Temperature sweep; FS: Frequency sweep.

**Fig 17:**
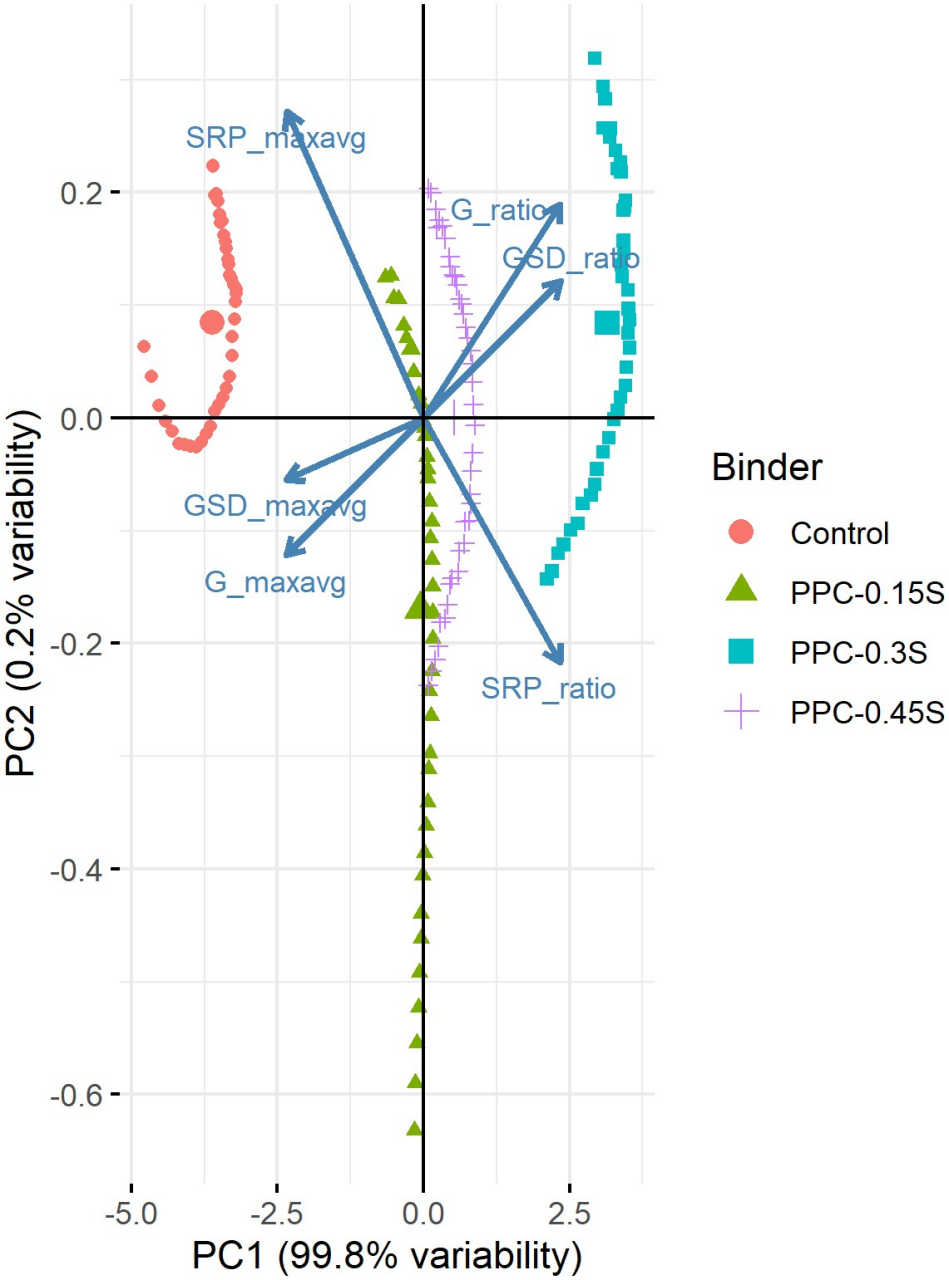
PCA biplot for temperature sweep.

**Fig 18:**
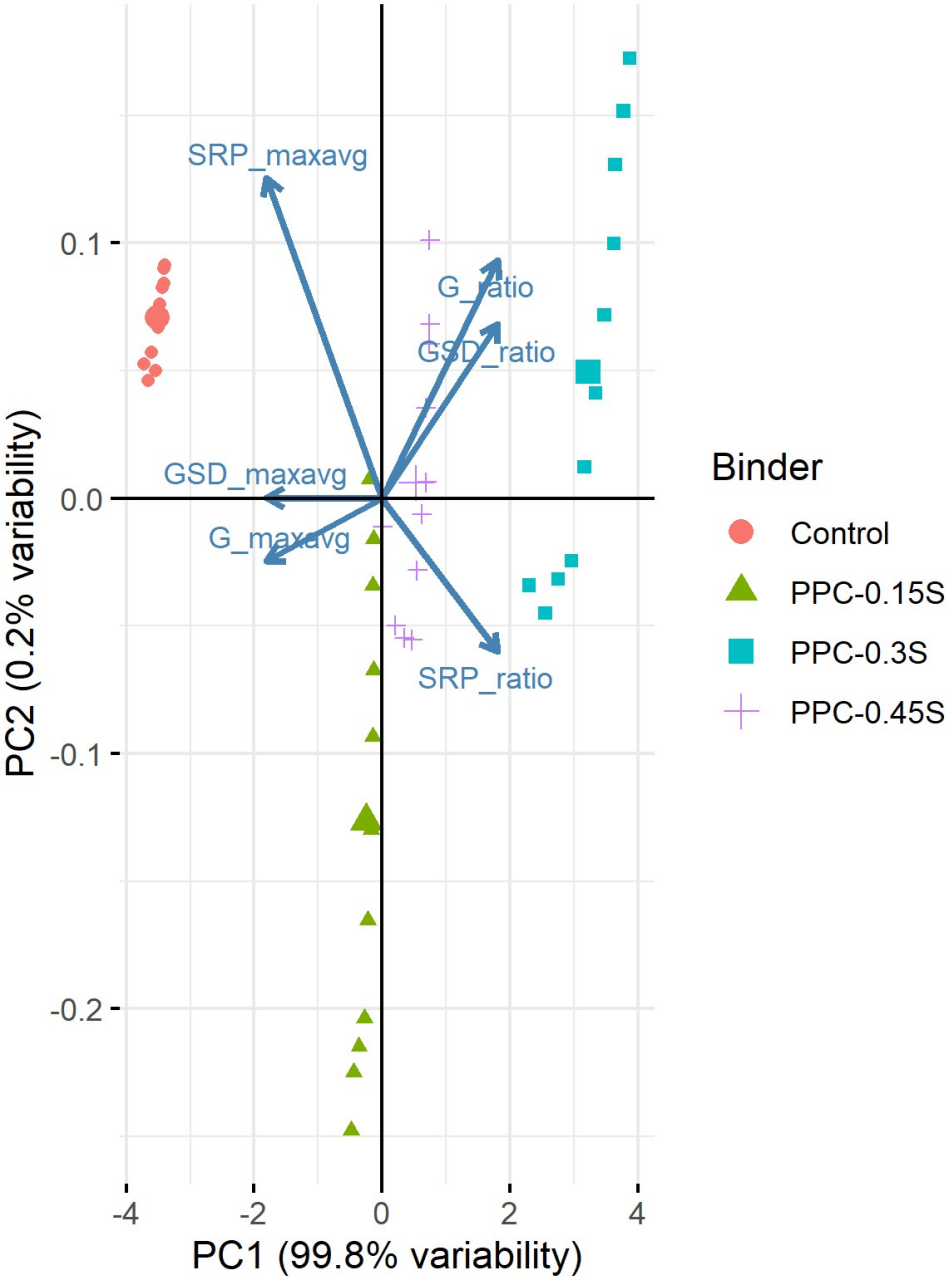
PCA biplot for frequency sweep.

Biplot is an important visual tool to understand the PCA output (Figs 17 and 18 for PCA based on temperature and frequency sweeps, respectively). On the *x*-axis, PC1 is plotted showing that it accounts for more than 99% of the variability. PC2 is plotted on the *y*-axis and shows it captures very small variability (~0.2%). Each arrow in Figs 17 and 18 represents the original variable (labelled adjacent to the arrow). The arrows for ratio formulated indices are in the positive direction of PC1, indicating that they have a positive correlation with PC1. On the other hand, the arrows for max-avg formulated indices are in the negative direction of PC1, indicating a negative correlation. Same observation can also be made from the inspection of signs on the loadings corresponding to the indices (Table 4). The points representing the control and binders with 0.15%, 0.3% and 0.45% sulfur are highlighted in different colors in Figs 17 and 18. It can be observed that the 0.3% binder plots far right on the positive side of PC1 (indicating higher values of ratio based indices and lower values of max-avg based indices), whereas the control binder plots far left on the negative side (indicating lower values of ratio based indices and higher values of max-avg based indices).

## Conclusions

The purpose of the present study was to evaluate the thermal storage stability of PPC modified binders using multiple dosages of sulfur. For this purpose, several rheology based separation indices were used in addition to the conventionally used softening point difference. The effect of ratio or maximum-average formulation of the indices was also analyzed. Further, FTIR spectroscopy was used as a qualitative approach to compare the chemical functionalities of the top and bottom section binders obtained from the storage stability test. Based on the results and analyses, the following conclusions are drawn:

- Asphalt binder modification even with a quite high dosage of plastic pyrolytic char (20%) showed good storage stability with sulfur when characterized through different rheological parameters and separation indices.
- Separation indices formulated in both forms (ratio form or maximum-average difference form) showed the same variation in storage stability performance. The variation of separation indices remained constant for temperatures 55 to 80°C and frequencies 1 to 100 rad/s.
- Addition of sulfur had marked improvement in storage stability of PPC modified binders. A sulfur dosage of 0.3% by weight of the binder showed the best separation performance among all dosages.
- Qualitative FTIR spectra analysis further showed that the closeness of spectral curves improved with the addition of sulfur.
- Principal component analysis showed that the ratio and maximum-average formulations had similar contributions to the first principal component accounting for more than 99% of the variability.
- The existence of an optimum separation performance at 0.3% sulfur content may be likely specific to the PPC and the base binder used in this study. Evaluation with more combinations of base binders and PPC sources are recommended.

Findings of the present study indicate that PPC modified binders with sulfur give a storage stable formulation. It is envisaged that the present findings would encourage further research to utilize the plastic pyrolysis char, a by-product generated from pyrolysis industries, for the production of modified asphalt binders and mixes.

In this study, the obtained rheological results were limited to the evaluation of unaged binders. A more detailed chemical characterization and evaluation of the rheology of short- and long-term aged PPC modified binders with different sulfur contents is recommended as a future scope of the work. Further investigations are also recommended to characterize performance of asphalt binders and mixes having modification through plastic pyrolysis char and sulfur.

## Supporting information

S1 File Data. (XLSX)
